# Splitting Things Apart to Put Them Back Together Again: A Targeted Review and Analysis of Psychological Therapy RCTs Addressing Recovery From Negative Symptoms

**DOI:** 10.3389/fpsyt.2022.826692

**Published:** 2022-05-12

**Authors:** Hamish J. McLeod

**Affiliations:** Institute of Health and Wellbeing, College of Medical Veterinary and Life Sciences, University of Glasgow, Glasgow, United Kingdom

**Keywords:** negative symptoms, motivation, apathy, psychological treatment, recovery

## Abstract

Negative symptoms have attracted growing attention as a psychological treatment target and the past 10 years has seen an expansion of mechanistic studies and clinical trials aimed at improving treatment options for this frequently neglected sub-group of people diagnosed with schizophrenia. The recent publication of several randomized controlled trials of psychological treatments that pre-specified negative symptoms as a primary outcome warrants a carefully targeted review and analysis, not least because these treatments have generally returned disappointing therapeutic benefits. This mini-review dissects these trials and offers an account of why we continue to have significant gaps in our understanding of how to support recovery in people troubled by persistent negative symptoms. Possible explanations for mixed trial results include a failure to separate the negative symptom phenotype into the clinically relevant sub-types that will respond to mechanistically targeted treatments. For example, the distinction between experiential and expressive deficits as separate components of the wider negative symptom construct points to potentially different treatment needs and techniques. The 10 negative symptom-focused RCTs chosen for analysis in this mini-review present over 16 different categories of treatment techniques spanning a range of cognitive, emotional, behavioral, interpersonal, and metacognitive domains of functioning. The argument is made that treatment development will advance more rapidly with the use of more precisely targeted psychological treatments that match interventions to a focused range of negative symptom maintenance processes.

## Introduction

Providing psychological therapies for paranoia and distressing hallucinations alongside pharmacotherapy and other medical treatments is now well-established in clinical guidelines (1) and there continues to be considerable innovation in the types of therapies being developed for positive symptoms [e.g. (2, 3)]. However, the negative symptoms of schizophrenia such as avolition-apathy and diminished expressive abilities have remained a major source of distress and arrested recovery that frequently present a significant treatment challenge (4, 5). Furthermore, surveys of people with a diagnosis of schizophrenia suggest that loss of emotional engagement and low motivational drive are a high priority for treatment (6) but at present there are very few effective psychological or pharmacological treatment options (7–9). This lack of progress in the development of viable treatments is particularly frustrating as earlier meta-analytic evidence suggested that even general CBTp led to medium effect size reductions on negative symptoms [*d* = 0.437, 95% CI: 0.171–0.704; (10)]. Also, it has become clearer that negative symptoms fluctuate more than previously assumed (11), leading to renewed hope that psychological interventions could be deployed to accelerate recovery. Reasons for cautious optimism can be drawn from recent meta-analytic evidence that suggests psychological treatments for negative symptoms can be beneficial, although the effects are less substantial when trial quality is factored in (12). To help accelerate the refinement of viable treatment packages this mini-review set out to analyze a range of negative symptom treatment trials conducted in the past decade with the aim of identifying ways that the targeting of treatments could be improved in future studies. Psychological treatment RCTs with the pre-specified aim of evaluating effects on negative symptoms were selected for review and analysis. The eligible papers were closely scrutinized, descriptive information was extracted, and themes and patterns across the studies were explored. Table 1 presents the key summary information from each paper with the emphasis placed on describing which negative symptoms were targeted, what therapeutic techniques were applied, the proposed mechanisms of therapeutic change, and the effects observed including acceptability and implementation outcomes such as attrition. Where the authors presented effect sizes these are reported to support description and comparison across studies.

**Table 1:** A descriptive summary of selected psychological treatment RCTs with negative symptoms specified as a primary or co-primary outcome 2009–2021.

**References**	**Sample Characteristics**	**Therapy Type, Format, and Dose**	**Outcomes Measured**	**Mechanism(s) of Change**	**Therapy Techniques**	**Observed Effects**	**Reported Primary Outcome Effect Size**
Klingberg et al. (13) Germany	198 people (44% female) with a SCID DSM-IV diagnosis of schizophrenia and at least one moderate severity PANSS negative syndrome factor and no PANSS positive or depression symptom score ≥ 6.	Two active treatment arms CBT vs. Cognitive Remediation delivered individually. Two phase modular CBT: Phase 1—psychoeducation, destigmatizing, and development of shared formulation. Phase 2—two out of five modules based on patient needs (e.g. support with planning, social skills) Twenty session over 9 months. Mean number of sessions: *CBT* = 16.6; *CR* = 13.7	Primary outcome was total PANSS Modified Negative Symptom Score (MNS; items N1, N2, N3, N4, N6, G7, G16) at 12 months post enrolment. Secondary outcomes were SANS subscale scores.	Social skills training Modification of self-defeating thought patterns Improvement of neurocognitive abilities	CBT: Shared formulation, improving self-understanding and acceptance, social skills training and feedback, Modifying expectations of failure. CR: Restitution and compensation based cognitive training focused on attention, memory, and executive functions.	No difference on primary outcome for CBT vs. CR. Both conditions improved.	Pre-post change on PANSS-MNS CBT *d* = −0.42 (95% CI: −0.70 to −0.13) CR *d =* −0.53 (95% CI: −0.82 to −0.25)
Grant et al. (14) USA	60 people with DSM-IV diagnosis of schizophrenia or schizoaffective disorder. At least moderate severity rating on 2 SANS subscales or marked severity on 1 subscale. Mean neurocognitive profile at least −1 SD below normal.	Individual outpatient sessions delivered weekly for 18 months. Average dose 50.5 sessions (range 16 to 81 sessions).	Clinician rated single item Global Assessment Scale (GAS) at post-treatment (18 months after randomization). Secondary outcomes were SANS subscale total scores and total SAPS score.	Modifying defeatist beliefs about reduced cognitive capacity, reduced behavioral competence, and reduced emotional competence [see Staring et al. (15)]	Collaborative goal setting, activity scheduling, behavioral experiments, challenging defeatist cognitions.	CBT treated patients showed greater improvement on Global Assessment of Functioning.	CT group GAS score *d =* −1.36 SANS Apathy *d* = −0.66.
Granholm et al. (16) USA	149 people with DSM-IV diagnosis of schizophrenia or schizoaffective disorder. No inclusion restrictions based on symptom profile.	Cognitive Behavioural Social Skills Training (CBSST) delivered in 36 weekly 2-h group sessions over 9 months. Monthly booster sessions were offered during 12-month post-treatment follow-up.	Primary outcome was self-reported functioning on the Independent Living Skills Survey (ILSS) at 9 months.	Asocial beliefs and defeatist performance beliefs (17)	Thought identification and change processes (e.g. 3c's), structured problem solving skills training, supported goal setting	CBSST arm showed significant improvements on the primary outcome. Retention was low across both the active and control treatment arms (54% retained at 9 months)	ILSS at 9 months *d*=0.55
Velligan et al. (18) USA	51 people with schizophrenia marked by clinically meaningful and persistent negative symptoms and no more than moderate positive symptoms, mild depression, and no significant movement disorder.	MOtiVation and Engagement (MOVE) Training—a manualized community delivered individual treatment. Sessions last for approximately 90 min once per week over 9 months.	Primary outcome was negative symptom assessed with the Negative Symptom Assessment 16 (NSA-16). The CAINS and BNSS were used in secondary analyses.	Negative symptoms are viewed as defense against the distress associated with judging the self as unable to cope. Maintenance cycles are established where atrophy of the capacity for initiation of behavior exacerbates loss of competence and self-confidence.	Five targeted domains of intervention including: Goal setting; social-cognitive skill rehearsal including social cue processing and social reciprocity; re-activation of leisure interests; anticipating and rating pleasure experiences; and linking of action plans to personally meaningful goals.	MOVE treated patients showed improvements on negative symptom measures at 9 months (post-treatment) compared to standard care.	
Priebe et al. (19) UK	275 people with an ICD diagnosis of schizophrenia and PANSS negative symptom subscale score ≥18.	20 sessions of body psychotherapy delivered in group format twice weekly for 10 weeks.	Primary outcome was PANSS negative symptom scale score immediately post treatment.	Gender (20) Group Climate (21)	Structured group tasks to strengthen awareness of the self, one's body, the boundaries between the self and others, and the use of movement as a mode of expression.	No difference between body psychotherapy and an active control (Pilates) on PANSS negative symptom score at post treatment. Improvements on expressive symptoms are small and not clinically meaningful.	
Mueller et al. (22) Switzerland	61 people with severe negative symptoms	Integrated Neurocognitive Therapy (INT)—a manualized CRT approach delivered over 15 weeks in group format. Organized into four therapy modules addressing 11 NIMH-MATRICS neurocognitive and social cognition problems.	Primary outcome was reduction of negative symptoms measured with the PANSS using Remission in Schizophrenia Working Group (RSWG) thresholds. Secondary outcomes included GAF and neurocognitive measures (e.g. Wisconsin Card Sorting Test).	Severe negative symptoms are argued to be under-pinned by neurocognitive deficits and problems with social cognition which may be targeted through structured remediation strategies.	Therapy techniques teach cognitive coping strategies (compensation), repeated skill practice (restitution), and *in vivo* application (generalization and “real-world” practice).	A significantly greater proportion of INT treated participants showed remission of severe negative symptoms at 3 months compared to standard care. Remission rate at 12 months showed a trend in favor of INT.	PANSS negative symptom score change at 3 months *d* = 0.31.
Favrod et al. (23) Switzerland	80 people with ICD diagnosis of schizophrenia (F20 or F25) and who scored at least 2 on the SANS Anhedonia scale.	8 x 60-min Positive Emotions Programme for Schizophrenia (PEPS) group treatment sessions for 5–8 patients.	Primary outcome was combined SANS avolition-apathy and anhedonia-asociality subscale scores.	Training of positive emotion regulation skills such as savoring, anticipation of pleasure, emotional expression training, challenging defeatist cognitions	Didactic and experiential delivery in group format. Verbally describing and sharing pleasant experiences	Primary outcome of combined SANS apathy-anhedonia scores improved in the treatment arm. Secondary outcomes of improved consummatory pleasure experiences also improved	Combined SANS apathy and anhedonia subscale scores *d =* −0.55.
Pos et al. (24) Netherlands	99 people in early phase of psychosis with DSM-IV-TR diagnosis of a schizophrenia spectrum disorder. Social withdrawal scores on PANSS and BNSS had to be at least in the mild range to be eligible for inclusion.	Combined group (8 sessions) and individual treatment (6 sessions) delivered over 3 months. 16 to 20 sessions.	Co-primary outcomes were Social Withdrawal scores on the PANSS and Brief Negative Symptom Scale score total and asociality scores.	Challenging defeatist beliefs and reducing self-stigma.	Psychoeducation, developing social goals, problem solving guidance	Both the intervention and control arm improved over time. Twenty percent attrition in active treatment vs. 30% in control.	
Buchanan et al. (25) USA	62 people with DSM-IV-TR diagnosis of schizophrenia or schizoaffective disorder. SANS asociality item needed to score ≥ 2 at baseline.	Four six session modules delivered over 24 weeks with repetition of each session to compensate for learning problems (total dose = 48 sessions). Treatment arm patients received intranasal oxytocin; controls received a placebo.	Birchwood Social Functioning Scale (BSFS) was the primary outcome at 24 weeks.	Enhancing social-affiliative information processing through exogenous oxytocin	Behavioral social skills practice, motivational interviewing, behavioral self-regulation strategy support, problem solving skills training	No post treatment between group differences in social functioning, defeatist beliefs, asocial beliefs.	-
Granholm et al. (26) USA	55 people with a DSM-IV diagnosis of schizophrenia or schizoaffective disorder with moderate to severe negative symptoms on the CAINS (total score ≥19). People with severe positive symptoms or depression were ineligible.	25 twice weekly 1 h group sessions for 12.5 weeks. Mean number of sessions attended was 8.65 (*SD* = 8.16 sessions) out of 25.	Total negative symptom scores (CAINS and SANS).	Modification of defeatist cognitions and augmentation of capacity to use psychological therapy through targeted cognitive remediation.	Cognitive-behavioural social skills training augmented with up to 8 sessions of cognitive remediation strategies focused on attention, prospective memory, and learning.	Main effect on SANS total at end of treatment (12 weeks) was mostly due to improvements on SANS Diminished Motivation score. Attrition was very high with 42% drop out in active treatment and 45% in standard care.	CAINS total *r* = −0.09; SANS total *r = –*0.22; SANS Diminished motivation *r* = −0.24.

## One Problem or Many? Subdividing the Negative Symptom Clinical Phenotype

One feature of this set of studies is that there is considerable heterogeneity in the clinical profile of people recruited into the trials and just about every study specifies a different constellation of primary and co-primary outcomes. This is an important issue as it is recognized that negative symptoms are more helpfully understood as comprising at least two separable sub-factors (27, 28) and that a very similar clinical phenotype can be seen when withdrawal and isolative behaviors are secondary to different underlying mechanisms such as positive symptoms or medication side effects (4, 29). Although nine of the included studies set some threshold for negative symptom severity as part of trial eligibility, only three clearly specified patient exclusion criteria based on the co-presence of positive symptoms or depressive features. These variations in method also extend to the ways that the primary outcomes were measured with seven studies using established negative symptom scales (e.g. CAINS, PANSS, SANS, BNSS) and the remaining three studies using measures of global functioning, social functioning, or independent living skills. There were also variations in practice across studies using established negative symptom measures as the outcome with some using a composite score of all negative symptoms and others selecting relevant subscale scores that indexed the negative symptom domain of interest [e.g. Favrod et al. (23) combined SANS avolition-apathy and anhedonia-asociality subscale scores as their primary outcome of the PEPS intervention programme]. As a result, the selected set of papers do not describe findings for a clearly delineated group of people with negative symptoms.

## What Works, On What and for Whom?

**Figure 1:**
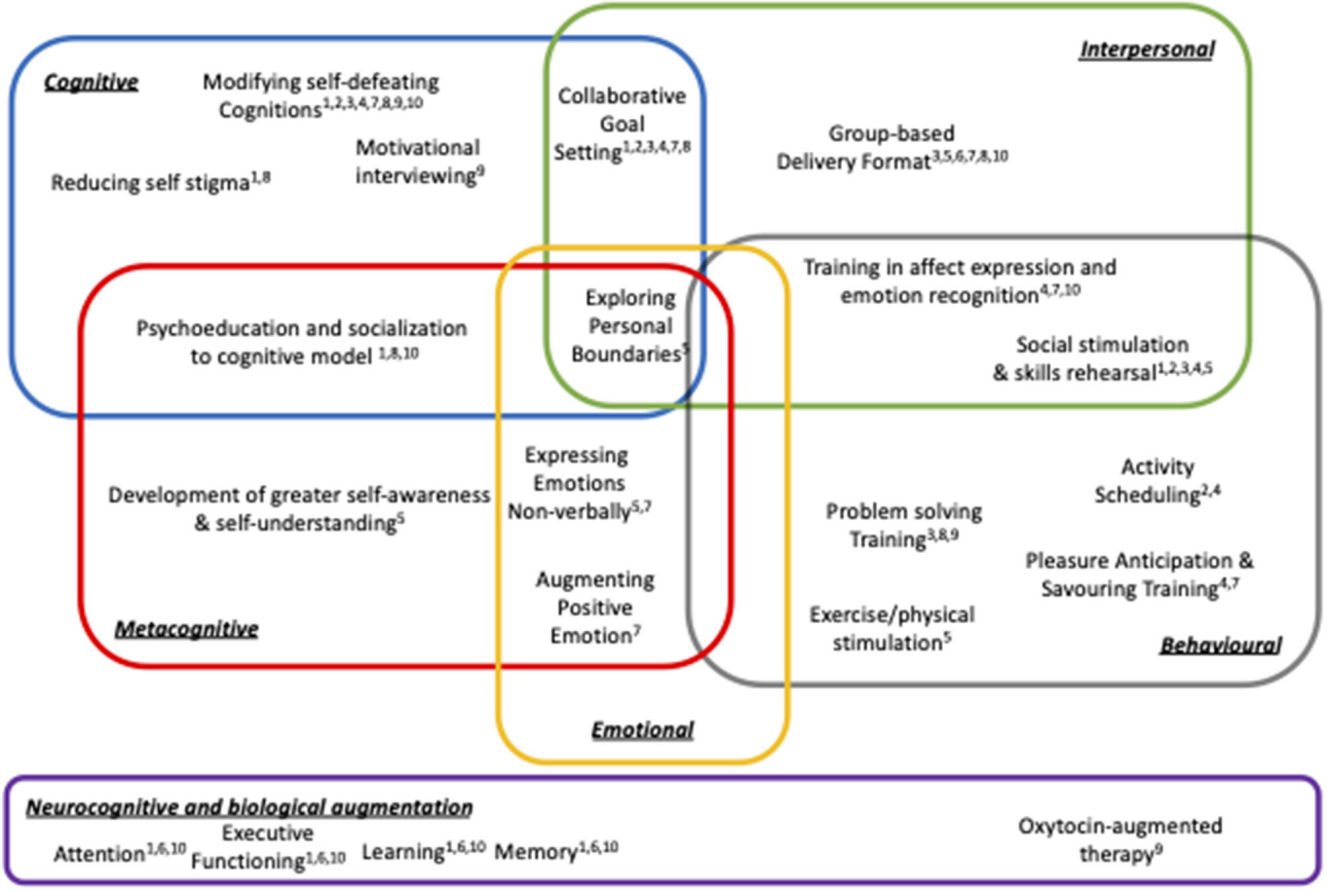
Schematic summary of treatment strategies across negative symptom focused RCTs (superscript numbers refer to papers listed in Table 1).

Six of the 10 studies returned results suggestive of at least some impact of the tested therapies on the targeted primary outcomes and the effect sizes reported are of a similar magnitude to those presented in previous meta-analyses (10). However, in several instances both the intervention and control arm patients showed improvements, possibly suggesting that for some people with negative symptoms giving *any* kind of supportive contact may be beneficial (30). The past 10 years has also seen a substantial increase in the studies testing one of the main tenets of the cognitive model of negative symptoms (31)—that self-defeating cognitions are a key cause and maintenance factor (32, 33). As described in Table 1 and depicted in Figure 1, helping people with negative symptoms to identify, challenge, and modify self-defeating beliefs is explicitly mentioned in eight of the 10 studies analyzed here. This is by far the most common strategy deployed across the studies. As highlighted in the mechanisms of change column of Table 1, two studies have supplementary analyses which suggest that modification of defeatist cognitions at least partially mediate treatment outcomes (15). The analysis of treatment mediators across other studies suggests that factors such as patient gender (20) and group climate (21) may also influence some treatment effects.

However, one of the key observations of this review is that understanding the mechanisms of change and the doses of therapy needed to produce beneficial effects is obscured by the extensive array of techniques, procedures, and therapy combinations that have been deployed to support people struggling to recover from negative symptoms. Figure 1 portrays this information in schematic form and shows that while the treatment protocols tested to date may share some features (e.g. attention to reducing defeatist cognitions), heterogeneity of treatment packages is the norm. It should be noted that the constellation of treatment techniques and the domains of therapeutic action depicted in Figure 1 does not fully capture all of the nuances and complex processes involved in the negative symptom therapy packages described. But, it does provide a framework for deconstructing and analyzing the psychological treatment methods that have been used to support people with negative symptoms. Splitting treatment packages into constituent parts provides one way of identifying testable hypotheses about plausible mechanisms of therapeutic change that can be then used to refine future therapy protocols.

## Splitting Things Apart

To advance our understanding of promising psychological treatment strategies for negative symptoms the therapeutic procedures described in each of the trials was “split apart” into constituent techniques. In some trials there was a clear link between the therapy techniques and the underlying theory of symptom formation and/or maintenance. For example, eliciting and challenging defeatist cognitions is a core feature of the dominant CBT model of negative symptoms and this leads to use of strategies such as belief modification and associated behavioral experiments. But, in other trials the link between the techniques and mechanisms of change were more opaque, or there were compound techniques that involved a mixture of potential change processes. Figure 1 depicts five overlapping categories of intervention that addressed cognitive, interpersonal, emotional, behavioral, and metacognitive domains. These are underpinned by a sixth neurocognitive/biological domain which has been introduced in a number of trials to convey how neural factors may provide a substrate that can constrain the potential for recovery (34, 35). By mapping the variety of therapy techniques reported across studies to this framework we can also see that some therapeutic strategies will require the operation of overlapping systems. For example, successfully exploring personal boundaries described in Body Oriented Psychotherapy may involve successful coordination of metacognitive, interpersonal, emotional and behavioral systems and a breakdown in any one domain may make it difficult for a person to fully capitalize on therapy. Other therapeutic strategies may be simpler to implement because they make less complex demands on the patient and can be structured and scaffolded by a therapist (e.g. activity scheduling). Hence, Figure 1 summarizes the candidate processes involved in supporting recovery from negative symptoms and tries to capture some of the reasons why the understanding of psychological treatment for negative symptoms is still very much a work in progress.

This approach to refining negative symptom treatments is warranted given the evidence that psychological treatments for positive psychotic symptoms have advanced through the use of causal manipulationist techniques that specify and modify psychological processes causally related to the clinical phenomenon of interest (2, 36). Currently the negative symptom treatment literature is dominated by multicomponent treatments, some elements of which are offshoots of experimental studies, but the specification of mechanistic targets is often incomplete. Next generation psychological treatment studies for negative symptoms are likely to benefit from a more explicit bottom-up development approach (37).

## Putting Things Back Together Again

A symptom rather than syndrome focus has been highly successful for psychological treatment research over the past 30 years (38) and in psychosis treatment studies the focus on specific symptoms has driven several therapy refinements (39). In some notable instances, treatment trials focused on discrete symptoms such as command hallucinations have produced some of the largest treatment effect sizes in the literature (40). When considering which negative symptoms to focus on in future treatment development, the current evidence suggests that at least the experiential and expressive subdomains should be treated as different types of problems in need of suitably tailored treatment approaches (41). The therapeutic value of more precisely matching treatments strategies to problem subtypes is beginning to be shown by meta-analytic results which suggest that CBT may be more effective for amotivation while cognitive remediation approaches may address problems of diminished expression (12). Future success in improving treatments will also be helped by following consensus guidelines that support the assessment and appropriate sub-classification of persistent, predominant, prominent, primary, and secondary negative symptoms (42). However, in taking specific symptom focused approach it is also important that future negative symptom treatment development does not lose sight of the whole person receiving care. In addition to “splitting things apart” to target specific symptoms we must also ensure that treatments also use person-centered formulation to help re-construct the fragmented self-experience that underpins schizophrenia (43). As highlighted in Figure 1, a number of the therapeutic strategies evident in existing treatment protocols are likely to be beneficial because they enrich the persons capacity to understand themselves, the boundary between the self and other people, and the operation of key experiences such as emotional self-regulation and the modulation of social interactions. Supporting these integrative processes is likely to be a necessary component of any successful psychosis intervention (44). This maps to the process of individual case formulation which has been shown to enhance the outcome of CBT for hallucinations (45) and may be particularly relevant to the improvement of interventions for negative symptoms. For example, some people with negative symptoms exhibit such severe disturbances of metacognitive functioning that they may find it extraordinarily difficult to even think about and reflect on their own mental state (46). Matching therapeutic techniques to both the reflective and neurocognitive capacities of the patient provides a way to help people with problematic negative symptoms regain the ability to link their ongoing experiences into the autobiographical narrative needed to support effective social and interpersonal functioning (47, 48). An important challenge for the next phase of negative symptom treatment development will be to convert the increasingly refined set of models used to understand specific negative symptoms into targeted and personalized therapies.

## Author Contributions

The author confirms being the sole contributor of this work and has approved it for publication.

## Conflict of Interest

The author declares that the research was conducted in the absence of any commercial or financial relationships that could be construed as a potential conflict of interest.

## Publisher's Note

All claims expressed in this article are solely those of the authors and do not necessarily represent those of their affiliated organizations, or those of the publisher, the editors and the reviewers. Any product that may be evaluated in this article, or claim that may be made by its manufacturer, is not guaranteed or endorsed by the publisher.
